# Lrp, a global regulator, regulates the virulence of *Vibrio vulnificus*

**DOI:** 10.1186/s12929-017-0361-9

**Published:** 2017-08-11

**Authors:** Yu-Chi Ho, Feng-Ru Hung, Chao-Hui Weng, Wei-Ting Li, Tzu-Hung Chuang, Tsung-Lin Liu, Ching-Yuan Lin, Chien-Jung Lo, Chun-Liang Chen, Jen-Wei Chen, Masayuki Hashimoto, Lien-I Hor

**Affiliations:** 1 Department of Microbiology and Immunology, College of Medicine, Tainan, 70101 Taiwan; 2 Institute of Bioinformatics and Biosignal Transduction, National Cheng Kung University, Tainan, 70101 Taiwan; 3 Department of Physics and Graduate Institute of Biophysics, National Central University, Taoyuan, 32001 Taiwan; 4 Center of Infectious Disease and Signal Transduction, National Cheng Kung University, Tainan, 70101 Taiwan; 5 Department of Molecular Medicine, National Cheng Kung University, Tainan, 70101 Taiwan; 6 Institute of Basic Medical Sciences, College of Medicine, National Cheng Kung University, Tainan, 70101 Taiwan

**Keywords:** *Vibrio vulnificus*, Spontaneous attenuated mutant, Lrp, Gene regulation, Virulence, Cytotoxicity, Chemotaxis, Iron-acquisition

## Abstract

**Background:**

An attenuated mutant (designated NY303) of *Vibrio vulnificus*, which causes serious wound infection and septicemia in humans, was isolated fortuitously from a clinical strain YJ016. This mutant was defective in cytotoxicity, migration on soft agar and virulence in the mouse. The purpose of this study was to map the mutation in this attenuated mutant and further explore how the gene thus identified is involved in virulence.

**Methods:**

The whole genome sequence of mutant NY303 determined by next-generation sequencing was compared with that of strain YJ016 to map the mutations. By isolating and characterizing the specific gene-knockout mutants, the gene associated with the phenotype of mutant NY303 was identified. This gene encodes a global regulator, Lrp. A mutant, YH01, deficient in Lrp was isolated and examined in vitro, in vivo and *ex vivo* to find the affected virulence mechanisms. The target genes of Lrp were further identified by comparing the transcriptomes, which were determined by RNA-seq, of strain YJ016 and mutant YH01. The promoters bound by Lrp were identified by genome footprinting-sequencing, and those related with virulence were further examined by electrophoretic mobility shift assay.

**Results:**

A mutation in *lrp* was shown to be associated with the reduced cytotoxicity, chemotaxis and virulence of mutant NY303. Mutant YH01 exhibited a phenotype resembling that of mutant NY303, and was defective in colonization in the mouse and growth in mouse serum, but not the antiphagocytosis ability. 596 and 95 genes were down- and up-regulated, respectively, in mutant YH01. Many of the genes involved in secretion of the MARTX cytotoxin, chemotaxis and iron-acquisition were down-regulated in mutant YH01. The *lrp* gene, which was shown to be negatively autoregulated, and 7 down-regulated virulence-associated genes were bound by Lrp in their promoters. A 14-bp consensus sequence, mkCrTTkwAyTsTG, putatively recognized by Lrp was identified in the promoters of these genes.

**Conclusions:**

Lrp is a global regulator involved in regulation of cytotoxicity, chemotaxis and iron-acquisition in *V. vulnificus*. Down-regulation of many of the genes associated with these properties may be responsible, at least partly, for loss of virulence in mutant NY303.

**Electronic supplementary material:**

The online version of this article (doi:10.1186/s12929-017-0361-9) contains supplementary material, which is available to authorized users.

## Background

*Vibrio vulnificus*, a gram-negative estuarine bacterium causing severe infectious diseases in humans worldwide [1–4], is the most invasive *Vibrio* species. Strains of *V. vulnificus* are divided into 3 biotypes, biotype 1, biotype 2 and biotype 3, by their biochemical properties, host ranges and epidemiological traits [5, 6]. In humans, this pathogen may cause fulminant septicemia with a high mortality rate and wound infections that can be as severe as necrotizing fasciitis, particularly in those with liver cirrhosis and hepatoma. Infection is usually acquired via ingestion of contaminated seafood, mostly raw oysters, or exposure of wounds to seawater/contaminated substances ([7, 8] for reviews).

Both the host factors, Iron-overloaded and immunocompromised conditions [9, 10], and bacterial virulence determinants contribute to the outcome of infection. Clinical *V. vulnificus* strains produce acidic capsular polysaccharides that prevent the bacterium from killing by complements and phagocytes. They also can acquire iron from the host via producing vulnibactin, a catechol-type siderophore that scavenges iron from various iron sources, and receptors for a variety of iron-containing substances. These properties have been demonstrated to be essential for the virulence of *V. vulnificus* in the mouse, which is a popular animal model for this pathogen [11–14]. This microorganism produces a few extracellular products, including the metalloprotease Vvp [15], cytolysin VvhA [16] and phospholipase Vpl [17]. A mutant deficient in Vvp, VvhA and Vpl is as virulent as the wild-type strain in the mouse (our unpublished data), indicating that they are all dispensable for virulence. The multifunctional autoprocessing repeats-in-toxin (MARTX), a cytotoxin secreted upon bacterium-cell interaction, is an important virulence factor required for bacterial survival during infection by protecting the organism from phagocytosis [18]. This toxin can also promote bacteria dissemination by causing intestinal tissue damage and inflammation [19]. Other virulence factors that have been studied include flagellum, pili, outer membrane protein OmpU, membrane-bound lipoprotein IlpA, and lysine decarboxylase. Nevertheless, except for the capsule, none of the above-mentioned factors has been shown to result in over 200-fold reduction in virulence if not produced.

We fortuitously isolated a spontaneous mutant that showed greatly reduced virulence in the mouse. In this study, the responsible mutation was mapped to the gene encoding an Lrp family member. We demonstrate that this global regulator controls, directly or indirectly, the expression of genes involved in a variety of biological activities, including those contributing to the virulence of *V. vulnificus*.

## Methods

### Bacterial strains, cell line, and culture conditions

The *V. vulnificus* and *Escherichia coli* strains (Table 1) were grown in Luria Bertani (LB) medium at 37 °C. The HeLa and RAW264.7 cells were cultured in Dulbecco’s modified Eagle’s medium with 10% fetal calf serum.

**Table 1:** Bacterial strains and plasmids used in this study

Strain/Plasmid	Description	Source/Reference
*Escherichia coli*		
S17-1λ*pir*	*Thi thr leu tonA lacY supE recA*::RP4–2 (Km::Tn*7*,Tc::Mu-1) lysogenized with λ*pir*	[21]
KA014	BL21(pMO08)	This study
WT29	ArcticExpress(pMO08)	This study
*Vibrio vulnificus*		
YJ016	Clinical isolate from blood	[30]
NY303–2	YJ016∆*vpl*∆*vvhA*	This study
NY303	Spontaneous attenuated mutant of NY303–2	This study
YH01	YJ016∆*lrp*, Cm^r^	This study
YH02	NY303–2∆*lrp*, Cm^r^	This study
YH03	YH01(p*lrp*)	This study
YH04	YH02(p*lrp*)	This study
YH05	NY303(p*lrp*)	This study
YH06	YJ016 containing lrp* cloned from NY303	This study
CH08	NY303–2 containing lrp* cloned from NY303	This study
CS9133	Clinical isolate (Biotype 1)	[21]
CH09	CS9133Δ*lrp*, Cm^r^	This study
CECT4999	Eel pathogen (Biotype 2)	[31]
CH10	CECT4999Δ*lrp*, Cm^r^	This study
KA023	YH01(pYU01)	This study
WT36	YJ016 integrated with P*lrp*-*lacZ* in *lacZ*	This study
WT37	YH01 integrated with P*lrp*-*lacZ* in *lacZ*	This study
WT38	YH06 integrated with P*lrp*-*lacZ* in *lacZ*	This study
HL128	YJ016∆*rtxA1*	[18]
CP212	YJ016∆*lacZ*	[44]
Plasmid		
pUC19	Cloning vector; Ap^r^	[65]
pCVD442	Cloning vector, *mob* RP4, *sacB*, and Ap^r^	[66]
pIT009	pJRD215 derivative with the Sm^r^ gene replaced by the MCS-containing *lacZ* gene cloned from pUC19	[31]
p*lrp*	pIT009 inserted with *lrp* cloned from strain YJ016	This study
pKCW01	pBBR1MCS4 inserted with *mazEF* cloned from pR99 at *Xba*I site	This study; [31, 67]
pYU01	pKCW01 inserted with *lrp* of strain YJ016, T7 terminator and His_6_-tag	This study
pMO08	pET30a(+)inserted with *lrp* cloned from strain YJ016	This study
pMO15	pCVD442 inserted with P*lrp*-*lacZ*	This study

*Km* kanamycin-resistance gene, *Tc* tetracycline- resistance gene, *Cm* ^*r*^ chloramphenicol-resistant, *Ap* ^*r*^ ampicillin-resistant, *Sm* ^*r*^ streptomycin-resistance, *MCS* multiple-cloning site

### Whole genome sequence determination and identification of mutations

The genomic DNA was extracted from an overnight bacterial culture in LB medium by a commercial kit (Promega) and then sent to the Center for Genomic Medicine, National Cheng Kung University (NCKU) for whole genome sequencing by SOLiD (ABI). BioEdit Sequence Alignment Editor, v.7.1.3.0 was used to compare the genome sequences of strain YJ016 and mutant NY303 for mapping the single nucleotide variations (SNVs) and short insertion/deletions in the mutant.

### Quantitative RT-PCR

Quantitative reverse transcription-polymerase chain reaction (qRT-PCR) was used to determine the transcriptional level of each gene. Briefly, total RNA was extracted from bacterial cells by RareRNA Kit (GenePure), and 1.5 μg of it was used to synthesize cDNA by reverse transcription with M-MLV reverse transcriptase (Promega). Real-time PCR was performed with StepOnePlus™ Real-Time PCR System (Applied Biosystem) and the threshold cycle (*C*_*T*_) value and relative quantification (RQ) level were determined by StepOne™ Software 2.1 (Applied Biosystem).

### Antibody preparation and Western bot hybridization

To prepare polyclonal anti-Lrp antibodies, Lrp was produced from the recombinant strain KA014 that expressed Lrp-his_6_ after induction at 30 °C for 6 h with 0.1 mM IPTG, and the proteins in cell lysate were fractionated by gel electrophoresis. The gel containing Lrp was excised, grounded, and mixed with complete (first injection) or incomplete adjuvant (other injections) for immunization of the 6–8 weeks old BALB/c mice by intraperitoneal injection. Mouse serum was collected after five consecutive injections every week, and stored at −80 °C. In Western blot hybridization, the proteins in total cell lysate were transferred onto a PVDF membrane after fractionation by SDS-polyacrylamide gel electrophoresis. The target proteins on the membrane were hybridized with relevant primary antibodies followed by secondary antibodies conjugated to horseradish peroxidase (HRP), and visualized by enhanced chemiluminescence. To prepare the anti-MARTX antibodies, the C-terminal his_6_-tagged ERM domain of *V. vulnificus* MARTX produced in *E. coli* NovaBlue (DE3) was purified by Chelating Sepharose Fast Flow (GE Healthcare). Purified ERM peptide was then used to generate polyclonal rabbit anti-ERM antiserum (AngeneBiotech, Taipei).

### Isolation of mutants, complemented strains and reporter strains

The ∆*lrp* mutants, each has the entire *lrp* gene replaced by the chloramphenicol acetyltransferase (CAT) cassette, were isolated by gene replacement. Briefly, the upstream and downstream regions flanking *lrp* were amplified from *V. vulnificus* YJ016 by PCR with the primer pairs Lrp-1/Lrp-2 and Lrp-3/Lrp-4 (Additional file 1: Table S1), cloned into pUC19 and then the CAT cassette was inserted between these two regions. The resultant plasmid was linearized with restriction enzyme and introduced into the wild-type strains by natural transformation [20]. The transformants were selected on the chloramphenicol (15 μg/ml)-containing LB plate. The deletion was detected by PCR and confirmed by DNA sequencing. For isolation of the lrp* mutants, the mutated *lrp* gene was amplified from mutant NY303 with the primer pair Lrp-1/Lrp-4 (Additional file 1: Table S1), which was then cloned into suicide plasmid pCVD442. The resultant plasmid was then used to generate the lrp* mutants by in vivo allelic exchange as described [21]. The lrp* mutants were screened by *Cla*I digestion of the PCR-amplified DNA fragments, in which the mutation disrupted the *Cla*I site, and confirmed by DNA sequencing. The complemented strains of the ∆*lrp* and lrp* mutants were obtained by transferring p*lrp*, a shuttle vector carrying the intact *lrp* gene amplified from strain YJ016 with primers Lrp-5 and Lrp-6 (Additional file 1: Table S1), from *E. coli* S17-1λ*pir* into these mutants by conjugation. To isolate *V. vulnificus* reporter strains that contain P*lrp-lacZ* in the chromosome, pMO15, a suicide plasmid carrying the *V. vulnificus lacZ* gene (amplified with primers JL109 and DC020) driven by the *lrp* promoter (amplified with primers Lrp-5 and DC017), was transferred from *E. coli* S17-1λ*pir* by conjugation. The transconjugants that had pMO15 integrated in *lacZ* were then selected on the ampicillin (100 μg/ml)-containing LB plate and confirmed by PCR with primers Lrp-5 and delacR (Additional file 1: Table S1).

### Migration on soft agar plate, electron microscopy, and swimming speed/flagellar motor switch analysis

10 μl of an overnight bacterial culture were inoculated onto a 0.3% agar plate and incubated at 37 °C for 8 h to show bacterial migration on soft agar. To examine the flagella, the bacteria were cultured in LB at 37 °C for 4 h, washed, adsorbed to a glow-discharged carbon-coated grid, stained with 2% uranyl acetate, and observed by transmission electron microscopy with JEM-1400 (JEOL). To analyze bacterial swimming behavior, 20 μl of 500 X diluted overnight culture were loaded into custom-built flow-chamber, and the bacterial swimming was observed under a phase contrast microscope (Ti-U, Nikon) with a 20 X objective. The images were taken at the middle of flow-chamber by a high-speed camera (GT1910, AVT) at 30 frames per second, and individual bacterial traces were analyzed by custom-written IDL program. Bacterial instantaneous swimming speed was calculated as the displacement between 2 frames divided by the time interval. The switching rate was calculated as the number of angle change larger than 90^o^ per second.

### Cytotoxicity and virulence assay

To assay cytotoxicity, the HeLa cells were coincubated with the bacteria in a 96-well microplate at 10 multiplicity of infection (moi) for 4 h, and the lactate dehydrogenase (LDH) released from lysed cells was estimated with a kit (Cyto Tox 96 non-radioactive cytotoxicity assay, Promega). In virulence assay, 6- to 8-week-old C3H/HeN mice were given subcutaneously a 10-fold serially diluted bacterial suspension in PBS. For each strain, four doses and five mice per dose were tested. The LD_50_ was calculated, as described previously [22], from the mortality at 72 h after challenge. All the animal experiments conducted in this study used the mice purchased from the animal center of College of Medicine in NCKU. The protocol of animal experiments was reviewed and approved by the Animal Ethics Committee of NCKU.

### Siderophore assay

Detection of vulnibactin in the culture supernatant was performed by Arnow test [23] with some modifications. Briefly, the culture supernatant collected from a 24 h bacterial culture at 25 °C in Synbase medium [24] was filtered by 0.45 μm syringe filter (Pall) and then 10-fold concentrated by lyophilization. Equal volume of 0.5 M HCl followed by equal volume of nitrite-molybdate reagent was added to the concentrate, and once the mixture turned yellow, equal volume of 1 M NaOH was added to stop the reaction. OD_517_ of the mixture was then measured.

### Colonization assay

2 × 10^6^ bacteria were inoculated into the air sac formed by subcutaneously injecting 1 ml of air on the back of a 6- to 8-week-old C3H/HeN mouse. 6 h after infection, the bacteria in the air sac were recovered with 1 ml PBS and enumerated by viable counts. The mouse was rendered neutropenic by injecting intraperitoneally with 3.75 mg of cyclophosphamide 3 days before infection.

### Phagocytosis assay

RAW264.7 cells were incubated with bacteria at a moi of 10 for 90 min at 37 °C. The cells were then lysed by 0.1% Triton X-100 and the total bacteria number in the well was enumerated by viable counts. The extracellular bacteria were killed by gentamicin (100 μg/ml) treatment for 30 min, and the intracellular bacterial number was enumerated after cell lysis. To determine the survival rate of internalized bacteria, the intracellular bacteria number was estimated at intervals after addition of gentamicin. The survival rate of the internalized bacteria was calculated by dividing the intracellular viable bacteria number at a given time point by that of 30 min after gentamicin treatment.

### Identification of Lrp target genes by transcriptome comparison

Total RNA was extracted by RNeasy Mini Kit (Qiagen) from a 2 h bacterial culture (2 × 10^6^/ml) in 80% mouse serum. The purity and concentration of the RNA solution were checked (≥20 μg total RNA, OD_260/280_ ≥1.8), and the RNA samples were sent to Genomics (New Taipei, Taiwan) for RNA-seq analysis with Illumina HiSeq™ 2000. Two samples were prepared for each of the wild-type strain and ∆*lrp* mutant, and the average of reads was used in comparison to identify the genes with ≥2 folds difference in mRNA levels between the two strains. Only those with a probability value ≥0.8 were considered reliable and, therefore, listed in Additional file 2: Table S2.

### Genome footprinting

The experiment we performed to identify the promoters bound by Lrp was modified from genome footprinting by high-throughput sequencing (GeF-seq) published by Chumsakul et al. [25]. We used *V. vulnificus* strain KA023, which overexpressed Lrp with a his_6_-tag at C-terminus and showed a wild-type phenotype, hoping that the candidate genes could be identified as many as possible. Formaldehyde (final concentration: 1%) was added to a 4 h-culture of *V. vulnificus* strain KA023 to cross link proteins with the bound DNAs. The bacterial cells were then broken up by sonication and the released DNA-protein cross-linking complexes were digested with DNase I (500 U/ml) to trim the bound DNAs. The treated cell lysate was subjected to immunoprecipitation with MagneHis™ Ni-beads to enrich the Lrp-his_6_-bound DNAs, and the protein-DNA complexes were heated at 65 °C to break the cross-links. The sequences of the collected DNAs were then determined by Genomics with Illumina HiSeq™ 2000.

### Electrophoretic mobility shift assay

The Lrp protein used in electrophoretic mobility shift assay (EMSA) was produced from strain WT29 after induction with 0.1 mM IPTG at 12 °C for 16 h, and purified by MagneHis™ Ni-Particles followed by dialysis. The promoter regions containing the *E. coli* consensus Lrp-binding sequences amplified from *V. vulnificus* YJ016 by PCR with the relevant primer pairs listed in Additional file 1: Table S1 were used as DNA probes. The mixture of DNA probe and Lrp-his_6_ was electrophoresed on a 6% native polyacrylamide gel. In simple EMSA, the DNA probe, with or without bound Lrp, was detected by ethidium bromide staining. In competitive EMSA, Lrp-his_6_ was mixed with the biotin-labeled DNA probe in the presence or absence of excess unlabeled DNA probe, and the unshifted and shifted DNA probes were transferred onto a positive-charged nylon membrane after electrophoresis. The bands containing the DNA probe were visualized with streptavidin-HRP followed by enhanced chemiluminescence.

### Computational analyses

To predict the Lrp-binding sites in *V. vulnificus*, the instances of *E. coli* Lrp-binding consensus sequences, yAGhAwATTwTdCTr (y: C or T; h: not G; w: A or T; d: not C; r: A or G) [26] and AGAATwwwATTCT [27], were searched in the genome of *V. vulnificus* YJ016 using TESS [28] allowing for three mismatches. Only the sites within intergenic regions were selected. To define the Lrp-binding motif of *V. vulnificus*, DNA sequences of ten *V. vulnificus* promoters shown to be bound by Lrp were analyzed by MEME [29]*.*

### Statistical analysis

The paired Student’s *t*-test (two-tailed) and one-way analysis of variance (ANOVA) along with Tukey’s test or two-way ANOVA with Bonferroni’s post test in the software Prism 5.01 were used for statistical analysis.

## Results

### Identification of the mutations in a spontaneous attenuated *V. vulnificus* mutant

**Fig. 1:**
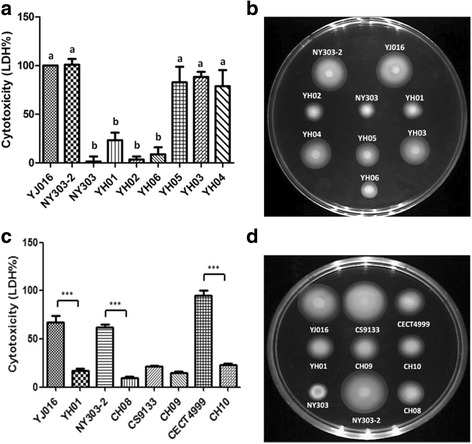
Migration on soft agar and cytotoxicity of the *V. vulnificus* strains. **a** and **c** HeLa cells were coincubated with the bacteria in a 96-well microplate at a moi of 10 for 4 h, and the cytotoxicity was then estimated by LDH assay. The percentage of cytotoxicity relative to that of strain YJ016 **a** or CECT4999 **c** is indicated. *n* = 3. **b** and **d** Bacterial culture were inoculated onto a 0.3% soft agar plate and incubated at 37 °C for 8 h to show the migration of each strain. YJ016: wild-type (biotype 1); NY303–2: YJ016Δ*vpl*Δ*vvhA*; NY303: spontaneous attenuated mutant derived from NY303–2; YH01: YJ016Δ*lrp*; YH02: NY303–2Δ*lrp*; YH03: YH01(p*lrp*); YH04: YH02(p*lrp*); YH05: NY303(p*lrp*); YH06: YJ016 lrp*; CH08: NY303–2 lrp*; CS9133: wild-type (biotype 1); CH09: CS9133Δ*lrp*; CECT4999: wild-type (biotype 2); CH10: CECT4999Δ*lrp*. Data in **a** were analyzed by one-way ANOVA along with Tukey’s test. *n* = 3. Bars that show no significant difference from each other are labeled with the same letter, and those showing significant difference (*P* < 0.05) are labeled with different letters. Data in **b** were analyzed by paired Student’s *t*-test. ***: *P* < 0.001

A unique ∆*vpl*∆*vvhA V. vulnificus* mutant (designated NY303) displaying a phenotype different from that of other ∆*vpl*∆*vvhA* mutants was isolated fortuitously. Mutant NY303 showed greatly reduced cytotoxicity and virulence (Fig. 1a; Table 2) while other ∆*vpl*∆*vvhA* mutants, represented by mutant NY303–2, were as cytotoxic and virulent as the parent strain, YJ016. This mutant remained noncytotoxic and weakly virulent after replacement of the deletions in *vpl* and *vvhA* with the intact genes (data not shown), indicating that the observed phenotype is associated with unknown mutation(s). NY303 was also defective in migration on soft agar plate (Fig. 1b) and exhibited altered outer membrane protein profile (data not shown), suggesting that the unknown mutation in NY303 might occurred to a global regulator. This mutant formed opaque colonies and was resistant to human serum (data not shown), however, indicating that its production of capsular polysaccharides was not affected.

**Table 2:** Virulence of the *lrp* mutants in the mouse

Strain	Description	LD_50_ (cfu/mouse)	Fold relative to YJ016
YJ016	Wild type	4.84 × 10^5^	1
NY303	Spontaneous attenuated mutant	1.99 × 10^8^	411
YH01	YJ016Δ*lrp*	1.45 × 10^8^	300
YH03	YH01(p*lrp*)	3.72 × 10^5^	0.77
YH06	YJ016 lrp*	5.46 × 10^6^	11
NY303–2	YJ016 Δ*vvh*Δ*vpl*	9.49 × 10^5^	2
CH08	NY303–2 -lrp*	7.00 × 10^7^	145
CS9133	Wild-type strain (biotype 1)	3.79 × 10^5^	0.78
CH09	CS9133Δ*lrp*	4.43 × 10^7^	92
CECT4999	Wild-type strain (biotype 2)	2.50 × 10^5^	0.52
CH10	CECT4999Δ*lrp*	6.32 × 10^7^	131

The bacteria were injected subcutaneously into the mouse, and the mortality was recorded within 72 h

To map the mutation(s) in mutant NY303, we determined the whole genome sequence of this mutant. By comparing the genome sequence of NY303 with that of YJ016 [30] and then confirming the mutations by sequencing the corresponding PCR products, we identified 6 SNVs, but no short insertion/deletions, in NY303. Among these SNVs, two were also present in mutant NY303–2, which showed the wild-type phenotype. The other four SNVs were located in the open-reading-frames annotated as leucine-responsive regulatory protein (Lrp, VV1321), ABC transporter permease (VV1373), UDP-glucose pyrophosphorylase (VV2955) and hypothetical protein (VVA0001), respectively.

### Lrp is an important regulator for cytotoxicity, migration on soft agar and virulence in the mouse

To determine which of the SNV-containing genes may be associated with the phenotype of NY303, we isolated and characterized the YJ016-derived mutants with single deletions in each of them. All of these genes, except the one encoding a hypothetical protein and being 1.66 kb downstream of the replication origin, were successfully deleted, and all of the resultant deletion mutants grew normally in LB medium. Among these mutants, only that with deletion in *lrp* (∆*lrp*), designated YH01, exhibited reduced cytotoxicity, migration on soft agar and virulence for the mouse, resembling mutant NY303 (Fig. 1a-b, Table 2). The other two mutants showed wild-type cytotoxicity and migration on soft agar (data not shown). We then complemented mutant YH01 with an *lrp*-carrying plasmid (p*lrp*), and found that all of these properties of the complemented strain were restored to the wild-type levels (Fig. 1a-b, Table 2). Deletion of *lrp* in strain NY303–2 also resulted in reduced migration on soft agar and cytotoxicity, which were restored to wild-type levels in the complemented strain (Fig. 1a-b). More, introduction of p*lrp* into mutant NY303 greatly improved its cytotoxicity and migration on soft agar (Fig. 1a-b). These results indicate that Lrp is essential for expression of virulence, and the mutation in *lrp* is linked with the phenotype of mutant NY303.

The Lrp mutation in mutant NY303 was located in the HTH domain, a DNA-binding motif (Additional file 3: Figure S1). To determine whether this point mutation (lrp*) alone is sufficient to cause the phenotype observed in mutant NY303, we isolated derivatives of strains YJ016 and NY303–2 (designated YH06 and CH08, respectively) that contained this mutation. Both mutants showed reduced cytotoxicity (Fig. 1a and c) and migration on soft agar (Fig. 1b and d) like NY303. They also exhibited lower virulence in the mouse compared to strain YJ016, although not as low as that of mutant NY303. In addition, the virulence of CH08 was lower than that of YH06 by about 13 folds (Table 2).

To exclude the possibility that the role of Lrp in virulence was strain-specific, we characterized the isogenic ∆*lrp* mutants, CH09 and CH10, derived from another biotype 1 strain, CS9133 [21], and a biotype 2 strain, CECT4999 [31]. Like mutant YH01, the virulence of CH09 and CH10 in the mouse was greatly reduced compared to their parent strains (Table 2). In addition, mutants CH09 and CH10 showed much less migration on soft agar and cytotoxicity, respectively, than their parent strains (Fig. 1c and d).

### The Δ*lrp* mutant is defective in survival in the mouse and growth in mouse serum, but not antiphagocytosis ability

**Fig. 2:**
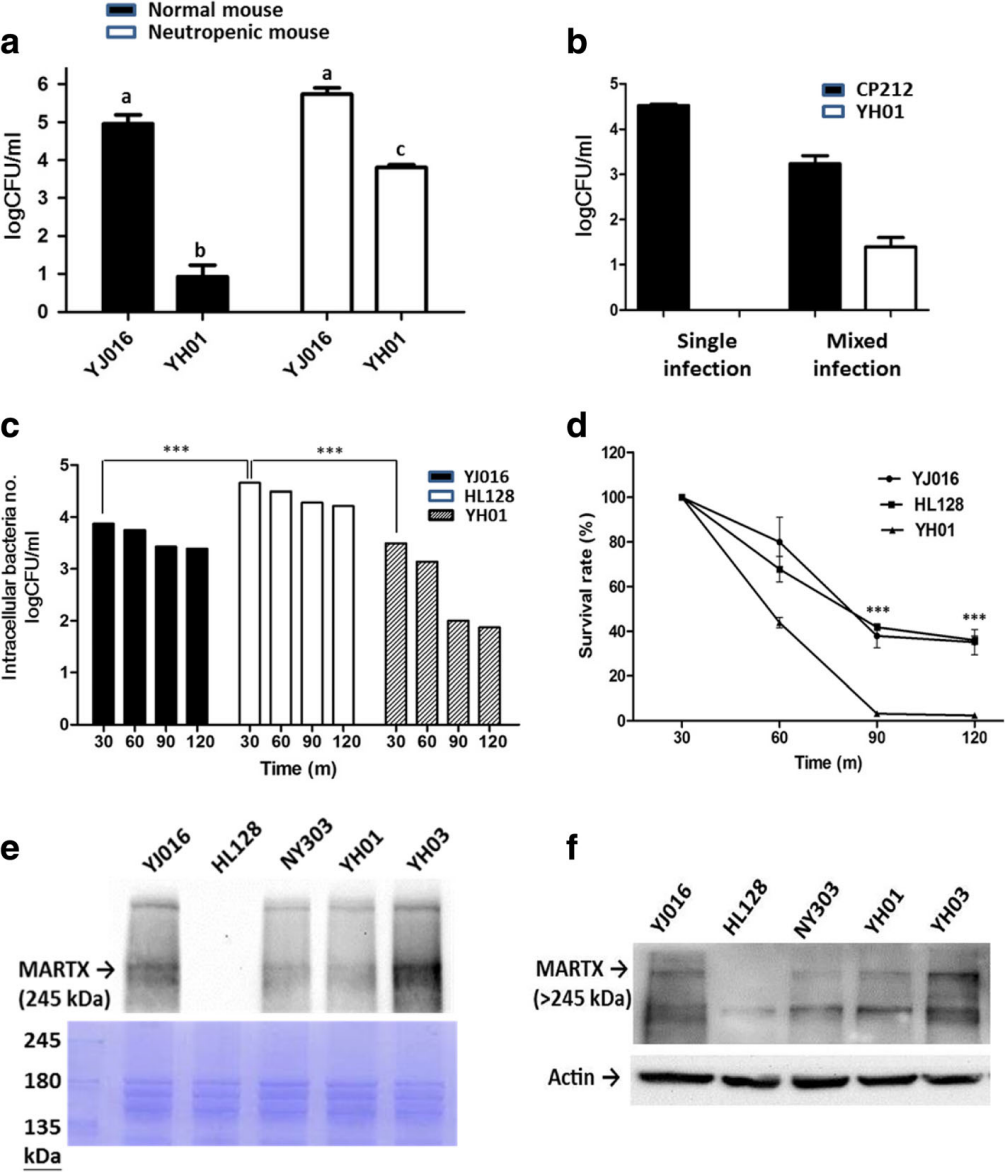
Colonization, antiphagocytosis activity and MARTX expression of the Δ*lrp* mutant. **a** The bacteria (2 × 10^6^ cfu) were injected into an air sac on the back of a normal or neutropenic mouse. The mice were sacrificed 6 h after infection, and the bacteria collected from the air sac were enumerated by viable counts. *n* = 3. YJ016: wild-type strain; YH01: Δ*lrp* mutant. The results of no significant difference analyzed by one-way ANOVA along with Tukey’s test are labeled with the same letters, and those of significant differences (*P* < 0.05) are labeled with different letters. **b** A mixture of equal numbers of mutant YH01 and wild-type strain CP212 (2 × 10^6^ cfu totally) were injected into the air sac on the back of a normal mouse. The mice were sacrificed 6 h after infection, and the viable bacteria collected from the air sac were enumerated by viable counts. *n* = 3. Strain CP212 was distinguished from mutant YH01 (*white* vs. *blue* colonies) on an X-gal-containing plate. **c** and **d** RAW 264.7 cells were cocultured with the bacteria at a moi of 10 for 30 min. At each time point, the intracellular bacteria number **c** was enumerated by viable counts after addition of gentamicin to kill the extracellular bacteria, and the survival rate of the internalized bacteria in **d** was calculated as described in ‘Phagocytosis assay’ of ‘Methods’. *n* = 3. YJ016: wild-type strain; HL128: Δ*rtxA1* mutant; YH01: Δ*lrp* mutant. The significance of difference was analyzed by t-test and two-way ANOVA with Bonferroni’s post test, respectively, for **c** and **d**. ***: *P* < 0.001 for the difference between YH01 and YJ016 or HL128. **e** and **f** Total cell lysates were prepared from the bacteria collected from a 4 h culture in LB broth **e** or RAW 264.7 cells cocultured with the bacteria at a moi of 10 for 1.5 h at 37 °C **f**. The proteins in the cell lysate were fractionated by electrophoresis on a 6% SDS-polyacrylamide gel and then subjected to Western blot analysis with anti-ERM antiserum. The upper panel in **e** is the result of Western blot analysis; the lower panel is the result of Coomassie blue stain of one of the duplicated gels to show that similar amount of proteins was loaded in each lane. β-actin was used as an internal control in **f**. YJ016: wild-type strain; HL128: Δ*rtxA1* mutant; NY303: spontaneous attenuated mutant; YH01: Δ*lrp* mutant; YH03: YH01(p*lrp*)

To understand how Lrp was involved in the virulence of *V. vulnificus*, we first compared strain YJ016 and mutant YH01 for their ability to colonize at the infection site. As shown in Fig. 2a, mutant YH01 was barely detected in the infected air sac, while strain YJ016 remained abundant at 6 h post inoculation. Depletion of neutrophils by cyclophosphamide improved colonization of mutant YH01, but not to the wild-type level (Fig. 2a). In addition, colonization of mutant YH01 was only slightly restored in the presence of strain YJ016 in mice infected by equal numbers of both strains (Fig. 2b).

The defect of mutant YH01 in colonization could be due to inability to resist clearance by phagocytosis and/or grow at the infection site. We examined the antiphagocytosis ability of mutant YH01, the MARTX-deficient mutant (HL128), which is defective in antiphagocytosis, and strain YJ016. As shown in Fig. 2c, the numbers of engulfed bacteria in macrophages were comparable between strain YJ016 and mutant YH01, and both were lower than that of mutant HL128. However, mutant YH01 was more rapidly killed in the macrophage compared to strain YJ016 or mutant HL128 (Fig. 2d). We also found that mutant YH01, like strain YJ016, could prevent phagocytosis of the co-incubated mutant HL128 by the macrophages (Additional file 4: Figure S2).

MARTX has been shown to mediate cytotoxicity and antiphagocytosis [18]. We therefore examined the amounts of this cytotoxin in bacterial lysate and infected macrophages. As shown in Fig. 2e and f, lower amounts of MARTX were detected in both the lysate of mutant YH01 and the macrophages infected by this mutant compared to those associated with strain YJ016.

**Fig. 3:**
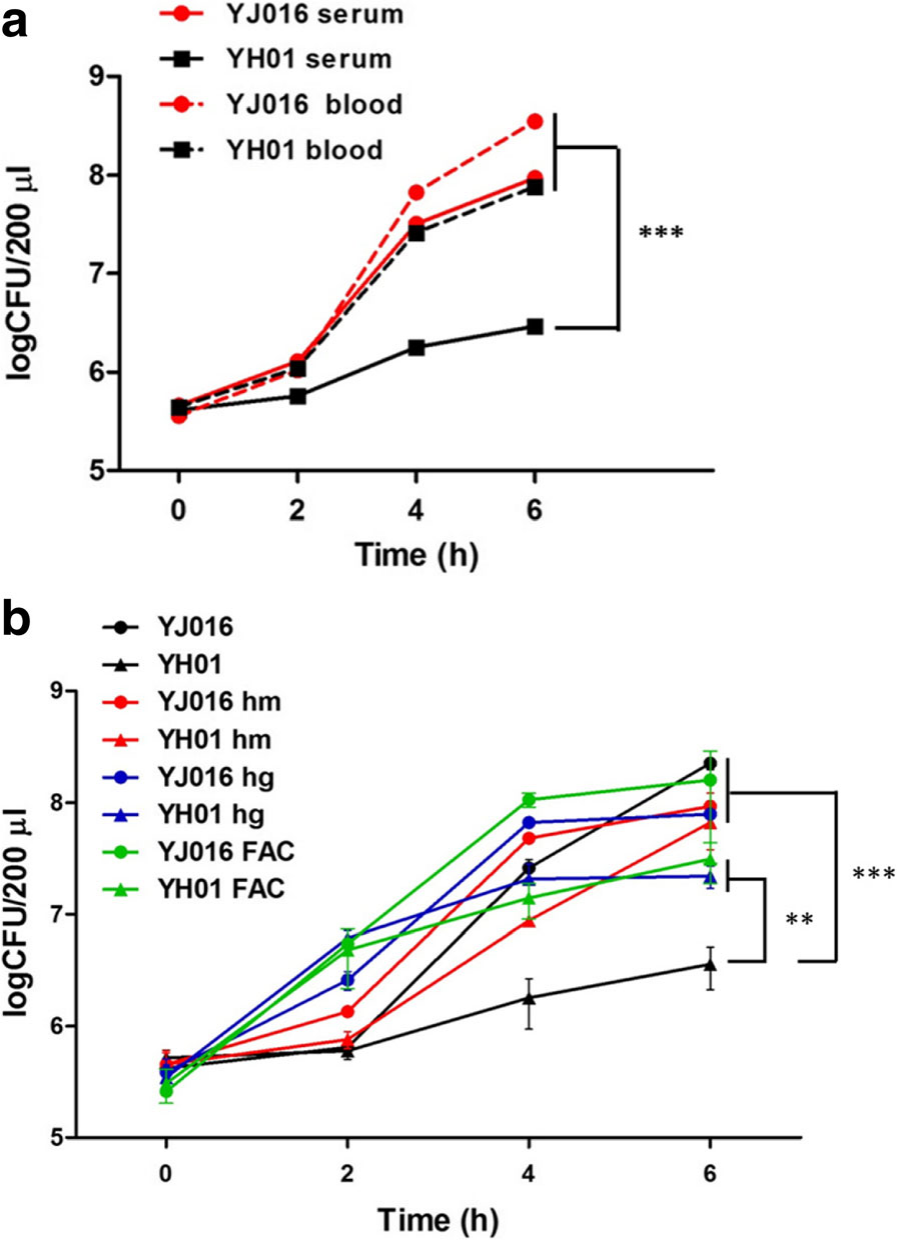
Growth of wild-type strain and Δ*lrp* mutant in mouse blood as well as serum with or without supplementation of iron sources. **a** The bacteria (6 × 10^5^ cfu/ml) were incubated in mouse serum or whole blood at 37 °C. **b** The bacteria (6 × 10^5^ cfu/ml) were incubated in mouse serum without supplementation or supplemented with hemin (hm; 10 μM), hemoglobin (hg; 10 μM) and ferric ammonium citrate (FAC:;200 μg/ml), respectively. The viable bacteria were enumerated by viable counts at the indicated time points. *n* = 3. YJ016: wild-type strain; YH01: Δ*lrp* mutant. The significance of difference was analyzed by two-way ANOVA along with Bonferroni’s post test. **: *P* < 0.01; ***: *P* < 0.001 for the difference between the results obtained at 6 h after incubation

We then examined the growth of mutant YH01 in mouse serum or whole blood to check whether it might be defective in growth in mouse tissue. As shown in Fig. 3a, mutant YH01 grew worse than strain YJ016 in the serum, but not whole blood, of the mouse. We suspected that the differential growth of mutant YH01 in serum and whole blood was because this mutant needed iron, which could be provided by heme released from the red blood cells, for growth. To test this, we traced the growth of mutant YH01 in mouse serum supplemented with hemoglobin, hemin or ferric ammonium citrate. As shown in Fig. 3b, the growth of this mutant was enhanced to the wild-type level in the presence of any of these iron sources.

### Identification of the target genes of Lrp

To explore how Lrp regulates the virulence of *V. vulnificus* in mice, we first identified the genes with altered expression levels in mutant YH01. We examined the *lrp* mRNA levels in strain YJ016 cultured in mouse serum and LB medium either with or without the iron-chelator, 2,2’-dipyridyl. We found that the *lrp* mRNA level decreased with time from 2 h to 6 h after incubation in each condition (Additional file 5: Figure S3a-c), and the highest level was detected in the mouse serum (Additional file 5: Figure S3d). In addition, no significant difference was observed between those cultured in LB in the presence and absence of the iron chelator. To analyze the transcriptomes, we incubated the bacteria in 80% mouse serum for 2 h and then determined the mRNA levels of each gene by RNA-seq (SRA accession number: SRP075529). By comparing the transcriptome of mutant YH01 with that of strain YJ016, we found that 95 and 596 genes were up- and down-regulated, respectively, by ≥2 folds (with a probability value ≥0.8) in mutant YH01 (Additional file 2: Table S2). A large fraction of the affected genes were predicted to be involved in the metabolic process (Additional file 6: Figure S4).

### Lrp is involved in the regulation of iron-acquisition and chemotaxis

**Fig. 4:**
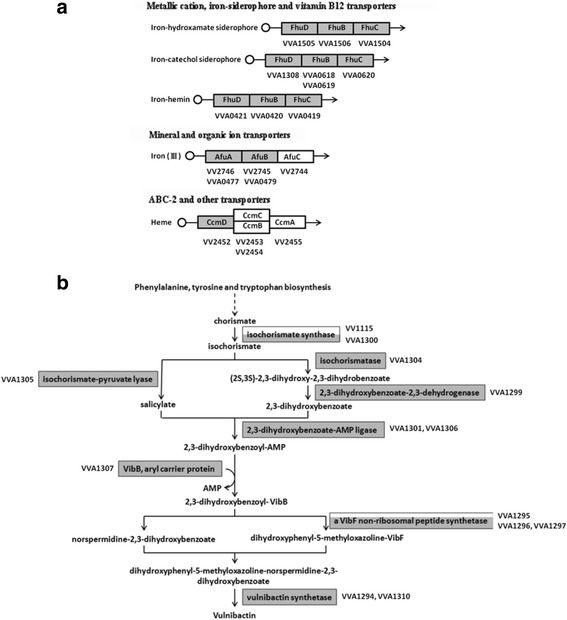
The genes involved in iron acquisition that were down-regulated in the Δ*lrp* mutant. The genes (presented by gene numbers) involved in iron transport **a** and vulnibactin biosynthesis **b** that were down-regulated in the Δ*lrp* mutant are indicated with *gray bars* on their putative products. The coverage of *gray* color in the bar reflects the % of genes annotated as a given enzyme that is affected

Notably, the genes encoding the heme receptor as well as some iron transporters and over 50% of the genes associated with the biosynthesis of vulnibactin were down-regulated in mutant YH01 (Fig. 4a and b). We performed qRT-PCR to check the mRNA levels of some of these genes, including VVA0781 (*hup*A), VVA1299, VVA1308, VVA1309 and VVA1310, in bacteria incubated in mouse serum and confirmed that they were all down regulated by more than 2 folds in mutant YH01 (Additional file 7: Figure S5a). We further performed Arnow test to detect the secreted vulnibactin and found that mutant YH01 produced much lower level of this siderophore than strain YJ016 (Table 3).

**Table 3:** Vulnibactin levels produced by *V. vulnificus* strains

Strain	Description	Arnow test	A_517_
YJ016	Wild type	+/−	0.018
YH01	YJ016Δ*lrp*	−	0.002
	Deferrated synbase medium	−	−0.006
	1 mM 2,3-DHBA	+	0.095

Arnow test is used for detection of catechol siderophores. -, negative; +/−, weakly positive (0.005 < A_517_ < 0.02); +, positive (A_517_ > 0.02)

Deferrated synbase medium and 1 mM 2,3-dihydroxybenzoic acid (2,3-DHBA) were used as negative and positive controls, respectively

**Fig. 5:**
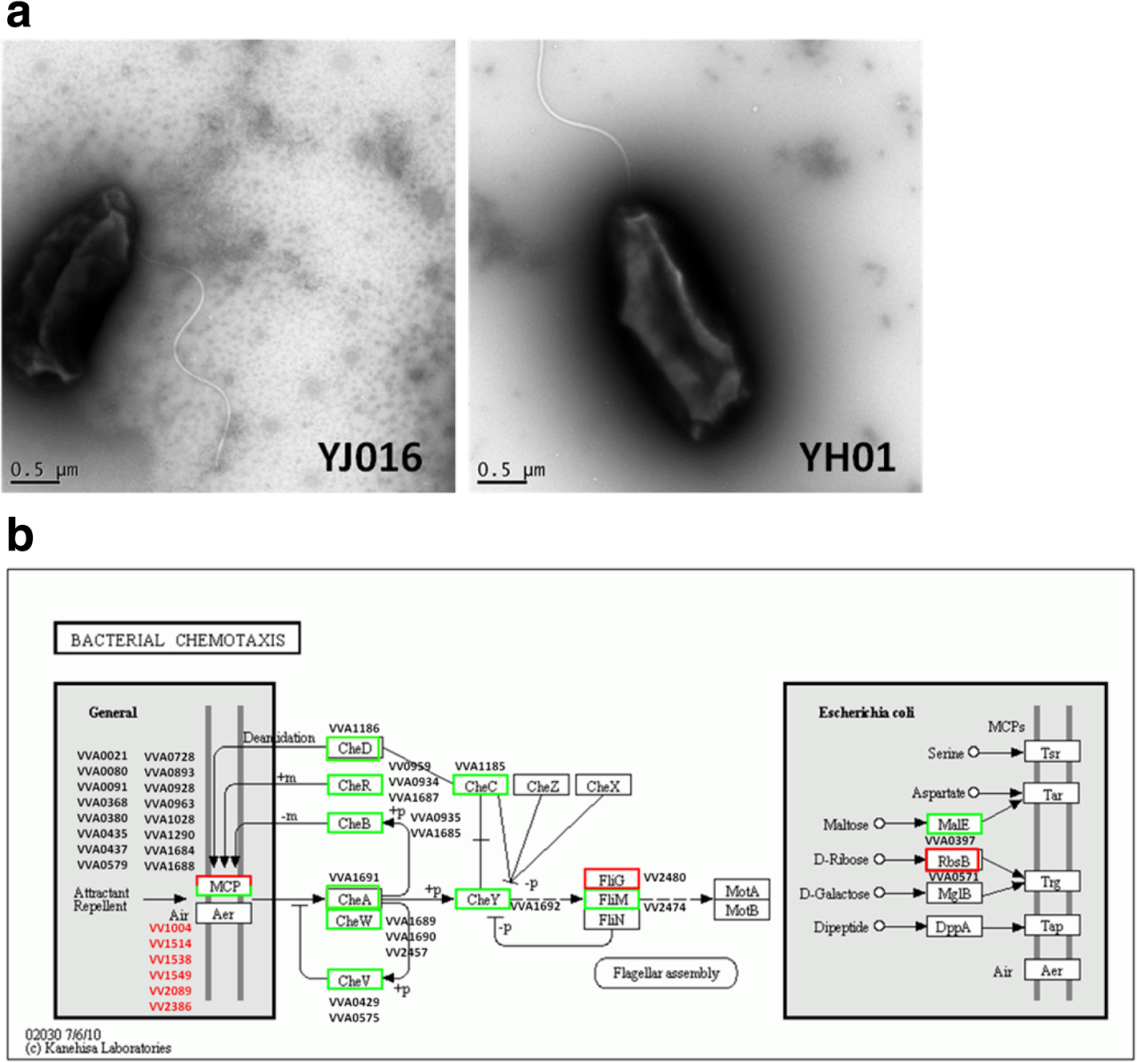
The appearance of flagellum and the chemotaxis genes with altered expression levels in the Δ*lrp* mutant. **a** Negative stain images of the flagella of wild-type strain (YJ016) and Δ*lrp* mutant (YH01) observed by transmission electron microscopy. **b** The putative chemotaxis genes with altered expression levels are indicated in the chemotaxis system of *V. vulnificus* illustrated in KEGG Map. The putative gene products of the genes (presented by gene numbers) down-regulated in the Δ*lrp* mutant are indicated with *green frame*; those of the genes up-regulated in the Δ*lrp* mutant are indicated with *red frame*. For those annotated as MCPs, some were down-regulated (labeled in *black*), and some others were upregulated (labeled in *red*); MCP is therefore indicated with a frame that is half red and half *green*

As decreased migration on soft agar could be due to the defect in motility or chemotaxis, we examined the flagellum of mutant YH01 and found that it appeared normal in width and length (Fig. 5a). We further observed bacterial motility in a flow chamber under a high-speed camera and found that the instantaneous swimming speed of YJ016, YH01 and YH03 (complemented strain of YH01) were 52.4 ± 16.3, 51.7 ± 13.9 and 51.8 ± 16.4 μm/s, respectively. On the other hand, the chemotaxis-dependent switching rates of YJ016, YH01 and YH03 were 0.58, 0.37 and 0.70 Hz, respectively, showing that mutant YH01 was defective in chemotaxis with a reduced switching rate. The transcriptome comparison data show that the transcriptional levels of only 4 genes (upregulated: *fliF* and *fliG*; down-regulated: *fliM* and *flgA*) among the over 30 genes involved in flagella assembly were altered in mutant YH01. However, a large number of chemotaxis-associated genes, including those annotated as methyl-accepting chemotaxis proteins (MCPs), CheA, CheW, CheY, CheC, CheD, CheV, CheR and CheB, were down-regulated in mutant YH01 (Fig. 5b). Some of these genes, including *VV2474, VVA0429, VVA0934, VVA1685, VVA1690* and *VVA1692*, were checked by qRT-PCR, and all were confirmed to be down regulated by more than 2 folds in mutant YH01 (Additional file 7: Figure S5b).

### Identification of genes directly regulated by Lrp

We conducted experiments modified from GeF-seq to identify the promoters bound by Lrp (SRA accession number: SRP075529). One thousand two hundred fifty one candidates were obtained. The *V. vulnificus* YJ016 Lrp protein shows 91% and 96% identity to full-length polypeptide and HTH domain, respectively, of the *E. coli* Lrp protein, suggesting that the two Lrps may bind to similar DNA sequences. Therefore, we also sought for promoters that contained the *E. coli* consensus Lrp-binding sequences by computational analysis, and identified 437 candidates.

**Fig. 6:**
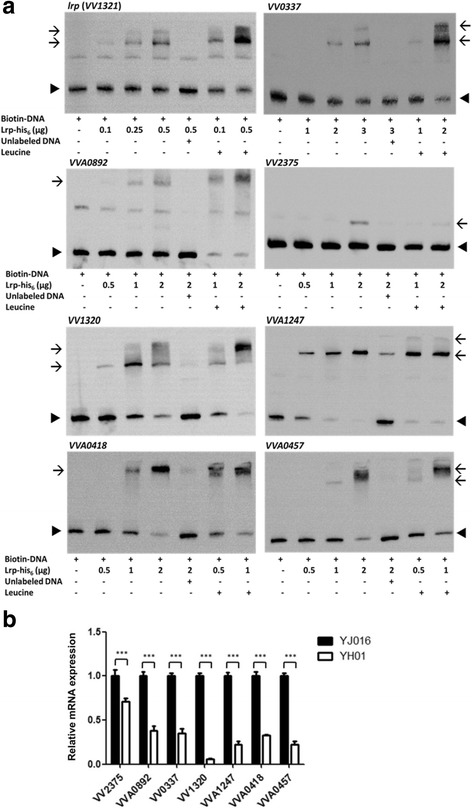
Lrp binding with the promoters of various genes and the mRNA levels of these genes in bacteria incubated in mouse serum. **a** Binding of biotin-labeled DNA probe with increasing amounts of Lrp (lanes 2–4) was competed with excess amount of unlabeled DNA (lane 5). The effect of leucine on binding of Lrp with DNA probe was also tested (lanes 6 and 7). **b** Total RNA was extracted from the bacteria cultured in 80% mouse serum for 2 h, and the mRNA level of each gene was estimated by qRT-PCR. *n* = 3. YJ016: wild-type strain; YH01: Δ*lrp* mutant. 23S rRNA was used as internal control. *Arrow head*: DNA probe; *Arrow*: shifted band corresponding to Lrp-bound DNA probe. ***: *P* < 0.001 analyzed by paired Student’s *t*-test

By comparing the result of bioinformatic prediction with those of modified GeF-seq and transcriptome comparison between the wild-type and ∆*lrp* strains described above, we found 72 and 218 common genes, respectively. Among them, we selected 40 that were annotated as virulence-related or transcriptional regulators to further confirm Lrp binding with their promoters by EMSA, and 8 of them showed positive results (Fig. 6a). They included *lrp* (*VV1321*) and genes associated with chemotaxis (*VVA1247*, *VVA0457* and *VV2375* annotated as CheY, chemotaxis protein and MCP, respectively), iron acquisition (*VVA0892* annotated as aromatic amino acid aminotransferase), capsule synthesis (*VV0337* annotated as capsular polysaccharide transport protein), amino acid metabolism (*VV1320* annotated as alanine dehydrogenase) as well as transcriptional regulation (*VVA0418*). Moreover, binding of Lrp with all of these promoters, except *VV2375*, was enhanced in the presence of 10 mM leucine. The expression levels of these genes other than *lrp* in mutant YH01 were significantly lower than those in strain YJ016 after incubation in 80% mouse serum (Fig. 6b).

The promoters of *tonB3* (*VV0250-VV0244*) and *cadBA* (*VV2382-VV2383*) have been previously demonstrated to be bound by Lrp [32, 33]. In these two promoters and the 8 Lrp-binding promoters we identified in this study, a 14-bp consensus DNA sequence, mkCrTTkwAyTsTG (m: A or C; k: G or T; r: A or G; w: A or T; y: C or T; s: G or C), was found.

### Autoregulation of Lrp

**Fig. 7:**
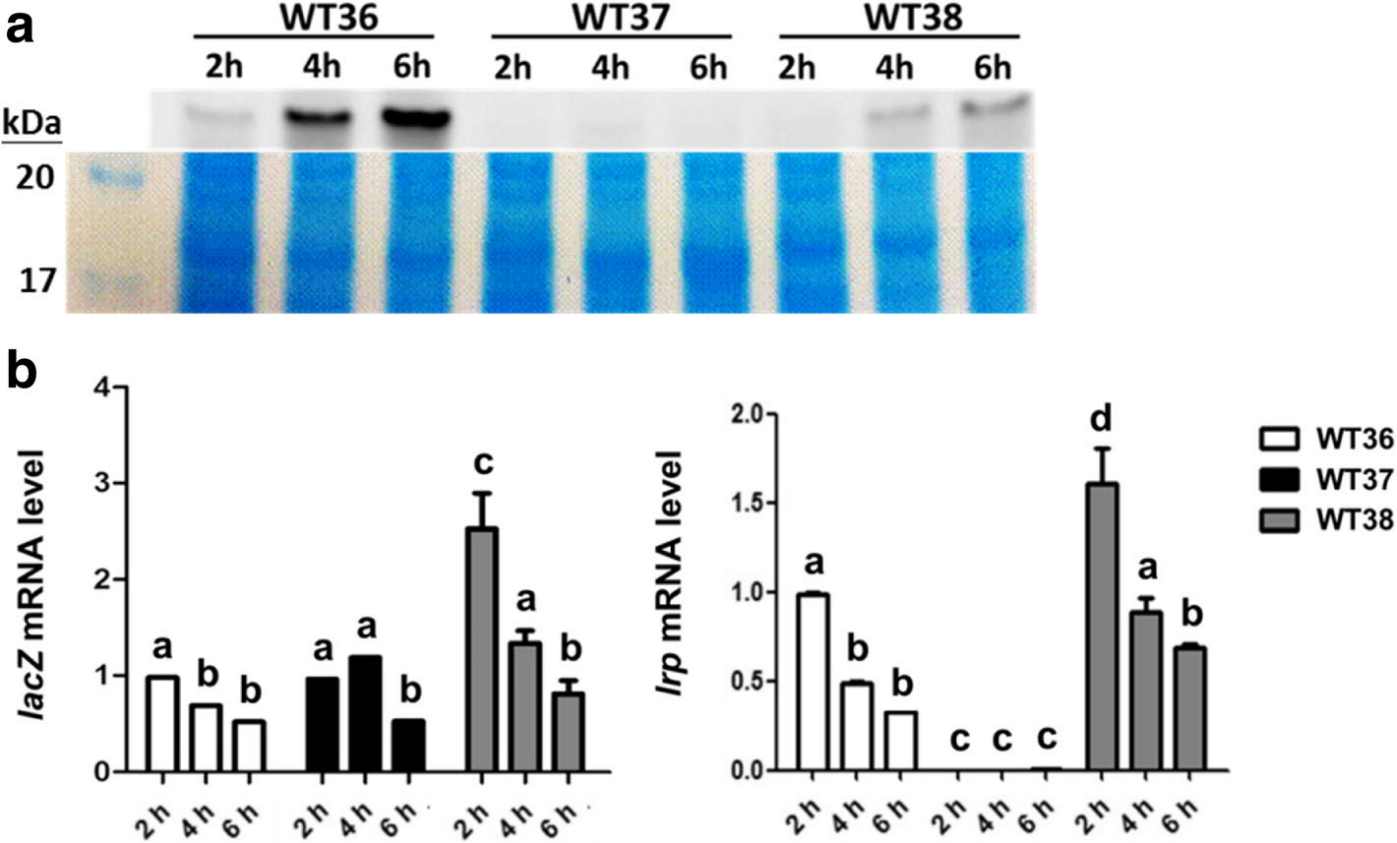
*lrp* promoter activity in the absence or presence of Lrp or Lrp*. **a** Lrp in total cell lysates of the various strains cultured in LB broth for the indicated periods was detected by Western blotting with anti-Lrp antibodies (*upper panel*). The lower panel is the result of Coomassie blue stain of one of the duplicated gels to show that similar amount of proteins was loaded in each lane for a given strain. **b** The mRNA levels of *lacZ* and *lrp* in bacteria cultured in LB broth for the indicated periods were determined by qRT-PCR*.* 23S rRNA was used as internal control. The ratio of each mRNA level relative to that of strain WT36 at 2 h is presented. *n* = 3. The results of no significant difference analyzed by one-way ANOVA along with Tukey’s test are labeled with the same letters, and those of significant differences (*P* < 0.05) are labeled with different letters. CP212: YJ016Δ*lacZ*;WT36: YJ016P*lrp*-*lacZ*;WT37: YJ016Δ*lrp*P*lrp*-*lacZ*;WT38: YJ016lrp*P*lrp*-*lacZ*

As Lrp could bind to the *lrp* promoter, suggesting that Lrp might regulate its own expression, we then investigated Lrp autoregulation by using *lacZ* as a reporter of the *lrp* promoter. As shown in Fig.7a, in the wild-type strain and lrp* mutant derivatives that have *lacZ* driven by the *lrp* promoter integrated in the chromosome, the amount of Lrp increased with time from 2 h to 6 h after being cultured in LB broth. However, both the *lrp* promoter activity (represented by *lacZ* mRNA level) and *lrp* mRNA levels decreased with time in either strain, with the lrp* mutant exhibiting highest levels of *lacZ* and *lrp* mRNAs at 2 h after incubation. The *lacZ* mRNA maintained at the basal level in the ∆*lrp* mutant up to 4 h followed by a slight decrease at 6 h after incubation (Fig. 7b).

## Discussion

*V. vulnificus* has long been noticed to cause rare but very severe infectious diseases in humans [34]. Although a number of virulence factors have been identified ([7, 8] for reviews), the virulence mechanism of *V. vulnificus* remains unclear. Victims of *V. vulnificus* infection usually acquire the pathogen from marine environment or contaminated seafood. It is well known that the coordinated expression of virulence factors in a bacterial cell is under strict control by a variety of regulatory networks that are activated upon detection of the host-related elements. A number of global regulators and regulatory systems, such as the cAMP-receptor protein (CRP) [35–37], Fur [38, 39], AphB [40], CsrA [41], IscR [42] and quorum-sensing signaling system [43, 44], have been shown to be involved in virulence regulation in this organism. In this study, we demonstrate that Lrp is another global regulator required for expression of virulence. Lrp does not seem to regulate virulence by affecting the expression levels of *crp*, *fur*, *aphB, csrA, iscR* or *luxO* (quorum-sensing regulator), because the mRNA levels of these genes were not significantly altered (<2-fold) in the ∆*lrp* mutant (data not shown).

Lrp is a transcription regulator found in a wide range of bacteria and archaea. In *E. coli* this regulator affects, either positively or negatively, about 10% of genes, most of which are involved in amino acid biosynthesis/catabolism/utilization, transport and pilus biosynthesis [45–48]. Besides, it plays an important role in bacterial growth in the nutrient limited environment [49]. Lrp has also been shown to regulate virulence genes in some pathogens, such as *Salmonella* Typhimurium [50], *Xenorhabdus nematophila* [51, 52], *V. cholerae* [53] and *Citrobacter rodentium* [54].

The *lrp* gene (VV1321) in *V. vulnificus* YJ016 encodes a protein of 164 aa, and is located between the genes encoding alanine dehydrogenase and FtsK, which is involved in cell division. This regulator has been shown to be involved in survival under stresses, like low pH, low temperature and high salt [55]. It is also involved in the regulation of *cadBA,* which is associated with acid tolerance [33], and the TonB3 system of unknown function [32] by direct binding to their promoters. But, not until now that Lrp is demonstrated to be also an important virulence regulator in this study.

The Lrp regulators are small proteins of about 15–18 kDa that contain the conserved DNA-binding helix-turn-helix (HTH) and effector-binding domains at N- and C-termini, respectively [45]. The mutation in *lrp* of the spontaneous attenuated mutant NY303 changes the 47th nucleotide from A to T, which results in substitution of Asp by Val at the 16th residue located in the HTH domain. Asp16 is highly conserved among the various archaeal and eubacterial Lrp homologs [56], and the Asp to Val mutation may affect Lrp binding with DNA. Introduction of this mutation (lrp*) into strains either with or without deletions of both *vvhA* and *vpl* resulted in reduced virulence, but not to the level of ∆*lrp* mutant (Table 2), suggesting that the Lrp* mutant protein may retain partial function. In an Lrp*-expressing strain, the *lrp* promoter activity was higher than that of a strain expressing the wild-type Lrp as was predicted from the Lrp’s role of a repressor, but the amount of Lrp* was lower than that of Lrp (Fig. 7a and b). This suggests that the lrp* mutation may lead to a decrease of not only the activity but also the amount of the mutated Lrp, probably resulting from inefficient translation or protein instability. The cytolysin (VvhA) and phospholipase (Vpl) were thought to be dispensable for virulence because mutant NY303–2, a ∆*vvhA*∆*vpl* mutant of strain YJ016, was as virulent as the parent strain (Table 2). However, here we show that deletion of both *vvhA* and *vpl* in an lrp* mutant greatly reduced the virulence, indicating that these two factors play some roles in virulence when the expression of several other virulence factors is reduced because of the mutation in Lrp.

Compared to the wild-type strain, the ∆*lrp* mutant is less able to colonize at the infection site. Unlike the MARTX-deficient mutant, the ∆*lrp* mutant did not colonize to the wild-type level in the neutropenic mice (Fig. 2a) or in the presence of the wild-type strain (Fig. 2b). More, the ∆*lrp* mutant exhibited wild-type resistance to engulfment by the macrophage (Fig. 2c), and protected the co-incubated MARTX-deficient mutant from phagocytosis (Additional file 4: Figure S2). Taken together, the defect of the ∆*lrp* mutant in colonization might not be due to its inability to prevent clearance by the phagocytes. Nevertheless, the ∆*lrp* mutant was more rapidly killed inside the macrophage than the wild-type or MARTX-deficient strain (Fig. 2d), probably due to its defects in resistance to the bactericidal mechanisms of the phagocytes.

It has been shown previously that both the cytotoxicity and antiphagocytosis activities of *V. vulnificus* are mediated mainly by MARTX. Intriguingly, the ∆*lrp* mutant, although is defective in cytotoxicity, retains the antiphagocytosis activity. Reduced MARTX amounts were detected in both the ∆*lrp* mutant and the macrophages it infected (Fig. 2e and f). It is likely that the amount of MARTX needed to cause cytotoxicity is higher than that needed to inhibit phagocytosis. The decreased MARTX levels in the ∆*lrp* mutant and cells infected by this mutant may result from down regulation of *rtxB* (VVA1034), *rtxD* (VVA1035) and *rtxE* (VVA1036), which encode the secretion system for MARTX. Down-regulation of *rtxB*, *rtxD* and *rtxE* by ≥2-folds (log2 ratios: −2.7, −1.9 and −1.8, respectively) was detected by transcriptome comparison between the wild-type and ∆*lrp* mutant, but because the probability values were all <0.8, they were not included in Additional file 2: Table S2.

The ∆*lrp* mutant grew poorly in mouse serum (Fig. 3a), but this was improved by supplementation of various iron sources (Fig. 3b). This suggests that the defect of this mutant in colonization may result from its poor growth under the iron-limited conditions existing in the host tissues, particularly upon infection. Consistently, the genes encoding the heme receptor as well as several iron transporters and more than half of those involved in vulnibactin biosynthesis were down-regulated in the ∆*lrp* mutant incubated in mouse serum (Fig. 4, Additional file 2: Table S2). At least one of them, VVA0892, predicted to encode the aromatic amino acid aminotransferase for synthesis of chorismate, was bound by Lrp at the promoter (Fig. 6a). Moreover, less vulnibactin was secreted by this mutant (Table 3). These indicate that Lrp could positively regulate the iron acquisition ability of *V. vulnificus*. Fur is known as a repressor for genes involved in iron acquisition, including vulnibactin biosynthesis [57]. In addition, SmcR was shown to repress the transcription of *vvsAB*, which encodes a nonribosomal peptide synthase required for vulnibactin biosynthesis, under iron-limited conditions [58]. We found in this study that SmcR was down-regulated by about 3 folds in the ∆*lrp* mutant incubated in mouse serum (Additional file 2: Table S2). Nevertheless, in this mutant the mRNA level of *vvsAB* was unaltered and the vulnibactin level was reduced, instead of increased as would be predicted from the role of SmcR as a repressor, suggesting a more predominant role of Lrp over SmcR in regulation of iron acquisition.

The reduced migration of the ∆*lrp* mutant on soft agar turned out to be a result of its defect in chemotaxis, but not motility by swimming. Consistently, the mRNA levels of only a few of the genes associated with flagellum assembly but many of those associated with chemotaxis, particularly those annotated as MCPs, are significantly down-regulated in the ∆*lrp* mutant (Additional file 2: Table S2 and Fig. 5b). It has been shown in *V. cholerae* that a non-chemotactic strain with clockwise biased flagellar rotation is attenuated for infection [59]. Whether chemotaxis may be important for the virulence of *V. vulnificus* awaits further investigation.

Eight genes, including *lrp*, that may be associated with virulence were shown to be bound by Lrp at the promoters (Fig. 6a), and their expression is down-regulated in the ∆*lrp* mutant (Fig. 6b). The *lrp* gene and 6 of the other 7 genes showed increased binding by Lrp and even super-shifted bands in the presence of leucine in EMSA, suggesting that leucine may play some role, such as stabilizing the Lrp oligomers [60], in the regulation of these genes. The putative consensus *V. vulnificus* Lrp-binding sequence, mkCrTTkwAyTsTG, deduced from the promoters of these genes is highly similar to that of *E. coli* [26, 27]. This is plausible as the Lrp, particularly its DNA-binding domain, of these two organisms share high identity in amino acid sequence.

We found that the transcription of *lrp* is higher in mouse serum than in the LB medium (Additional file 5: Figure S3), probably because compared with the LB medium mouse serum is more mimicking a state of famine. In addition, the *lrp* mRNA levels in LB medium with or without iron chelator were comparable, suggesting that *lrp* may not be regulated by iron concentration. We also demonstrated in a reporter strain that, like those in *E. coli* [61], *Agrobacterium tumefaciens* [62] and *Salmonella* Typhimurium [63], the Lrp of *V. vulnificus* is negatively autoregulated (Fig. 7b and c). Surprisingly, the *lrp* promoter activity was not increased in the ∆*lrp* mutant, compared to that in the wild-type strain (Fig. 7b). It has been shown in *E. coli* that *lrp* is positively regulated by GadE [64]. Although no activator of *lrp* has been reported in *V. vulnificus* so far, it is possible that an activator of *lrp* was down-regulated in the absence of Lrp, and this resulted in only basal level of *lrp* transcription.

## Conclusions

We demonstrate that Lrp, a global regulator, is an important virulence regulator of *V. vulnificus*. The isogenic Lrp-deficient mutant of a clinical strain is defective in cytotoxicity, chemotaxis, growth in mouse serum, and virulence in the mouse. Further, compared to the wild-type strain, many of the genes involved in secretion of MARTX cytotoxin, chemotaxis and iron-acquisition are down-regulated in the Lrp-deficient mutant. Eight virulence-associated genes, including *lrp* itself, have been shown to be bound by Lrp at their promoters, and a consensus Lrp-binding sequence, mkCrTTkwAyTsTG, can be defined. Lrp is also a repressor for its own gene. Collectively, our data suggest that Lrp might regulate the virulence of *V. vulnificus* by, at least partly, directly or indirectly activating some of the genes involved in cytotoxicity, chemotaxis and iron-acquisition during infection. These findings pave the way for uncovering the regulatory network of virulence and identifying novel virulence genes in *V. vulnificus*.

## Additional files

Additional file 1: Table S1. Primers used in this study. (DOCX 19 kb)

Additional file 2: Table S2. Lrp target genes identified by transcriptome comparison. (XLSX 427 kb)

Additional file 3: Figure S1. Location of point mutation in *lrp* of *V. vulnificus mutant* NY303. (DOCX 72 kb)

Additional file 4: Figure S2. Phagocytosis of various *V. vulnificus* strains by RAW 264.7 cells after mixed infection. (DOCX 55 kb)

Additional file 5: Figure S3. The mRNA levels of *lrp* in the wild-type strain incubated in various media. (DOCX 158 kb)

Additional file 6: Figure S4. The distribution of Lrp target genes in different functional categories. (DOCX 334 kb)

Additional file 7: Figure S5. The mRNA levels of genes predicted to be involved in iron-acquisition and chemotaxis in the wild-type strain and Δ*lrp* mutant. (DOCX 219 kb)

## Abbreviations

ABC: ATP-binding cassette
cfu and CFU: Colony-forming unit
HRP: Horseradish peroxidase
LB medium: Luria-Bertani medium
LDH: Lactate dehydrogenase
Lrp: Leucine-responsive regulatory protein
moi: Multiplicity of infection
qRT-PCR: Quantitative reverse transcription-polymerase chain reaction
SNV: Single nucleotide variation
UDP: Uridine diphosphate
X-gal: 5-bromo-4-chloro-3-indolyl-β-D-galactopyranoside
