# Supplementary material for: Lrp, a global regulator, regulates the virulence of Vibrio vulnificus
Source: J Biomed Sci. 2017 Aug 11;24:54. doi: 10.1186/s12929-017-0361-9 (PMC5554404; doi:10.1186/s12929-017-0361-9)
Supplement: Supplementary file 1 — Primers used in this study. (DOCX 19 kb) [file 12929_2017_361_MOESM1_ESM.docx]

Table S1. Primers used in this study.

|  | Sequence (5'-3') ^a^ | | | Purpose |  |
| --- | --- | --- | --- | --- | --- |
|  |  |  |  |  |  |
| Lrp-1 | | CAACAGCGGCACCTGGCTTC | Isolation of Δ*lrp* mutants | | |
| Lrp-2 | | CTAGATCCTTGGACGGCT |  |  |  |
| Lrp-3 | | GGAGCTCGCAAACCAACCAACTTGTG | Isolation of Δ*lrp* mutants | | |
| Lrp-4 | | GGAGCTCGTGGCTGAGCTTTAGGTTC |  |  |  |
| Lrp-5 | | GGGAATTCGTAGGAAGAATGGATGCG | Cloning of *lrp* gene | | |
| Lrp-6 | | CGGAATTCGCTCGGATTCATAAGTCG |  |  |  |
| Lrp-11 | | CCCTCAATACCTAGACGC | Detection of *lrp* gene or *lrp* mRNA | | |
| Lrp-12 | | CGGTAAGCACCCATATCAG |  |  |  |
| DC017 | | GGGAAGCTTTTATTCCACCTTATTACT | Cloning of P*lrp* in combination with Lrp-5 | | |
| JL108 | | CCCAAGCTTATGACTGCATTTTCAGAA | Cloning of 5’ part of *lacZ* (*VVA0165*) (*lacZ*’) | | |
| JL109 | | TATGAGCTCCACTTTACGGAAACCCAC |  |  |  |
| DC020 | | ATAGAGCTCGTAGGAAGAATGGATGCI | Cloning of P*lrp-lacZ’* in combination with JL109 | | |
| delacR | | CCACGTTCCATTCCATCAGC | Confirming pMO15 integration into *lacZ* | | |
| DC018 | | GTAGGAAGAATGGATGCG | DNA Probe of *VV1321* (*lrp*) promoter in EMSA | | |
| DC019 | | CTAGATCCTTGGACGGCT |  |  |  |
| DC031 | | GCCAACGTTGTGTGCATCTCA | DNA Probe of *VV2375* promoter in EMSA | | |
| DC032 | | GAAGCCGGCATACATCCTTTT |  |  |  |
| DC035 | | GCGGATCGTTACGAAAAGCAA | DNA Probe of *VVA0892* promoter in EMSA | | |
| DC036 | | CAGGGAGAGAATTCGACACGG |  |  |  |
| DC049 | | TCCTTGGACGGCTTTTTGTAG | DNA Probe of *VV1320* promoter in EMSA | | |
| DC050 | | CATCGTCTGAAAAACCGATGC |  |  |  |
| DC055 | | CGAGAATAGCGCTACAGATTGGAG | DNA Probe of *VVA1247* promoter in EMSA | | |
| DC056 | | CAATCCAACAAAACGATACACCC |  |  |  |
| DC083 | | ACCGGATTTCACCTTTTACTTACTTAG | DNA Probe of *VVA0418* promoter in EMSA | | |
| DC084 | | AACAACGGCATCCATAATTTTCTG |  |  |  |
| DC087 | | CAGTTGGCTAGGTGACCCTCG | DNA Probe of *VVA0457* promoter in EMSA | | |
| DC088 | | AACCGACGTAAAAGCGAATGC |  |  |  |
| DC137 | | CTCAGTGGTGTCATAAAACGTCAGTACT | DNA Probe of *VV0337* promoter in EMSA | | |
| DC138 | | AACACCATTTCTTTCTAACGCTTTTG |  |  |  |

^a^ The restriction sites are marked with underlines.
