# Supplementary material for: Lrp, a global regulator, regulates the virulence of Vibrio vulnificus
Source: J Biomed Sci. 2017 Aug 11;24:54. doi: 10.1186/s12929-017-0361-9 (PMC5554404; doi:10.1186/s12929-017-0361-9)
Supplement: Supplementary file 3 — Location of point mutation in lrp of V. vulnificus mutant NY303. (DOCX 72 kb) [file 12929_2017_361_MOESM3_ESM.docx]

A


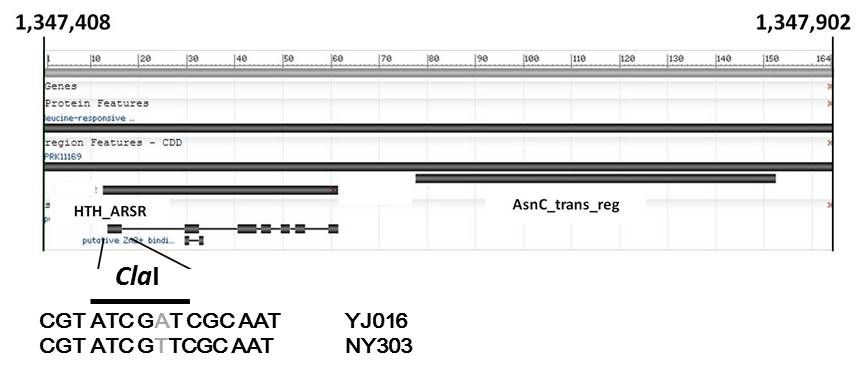


B


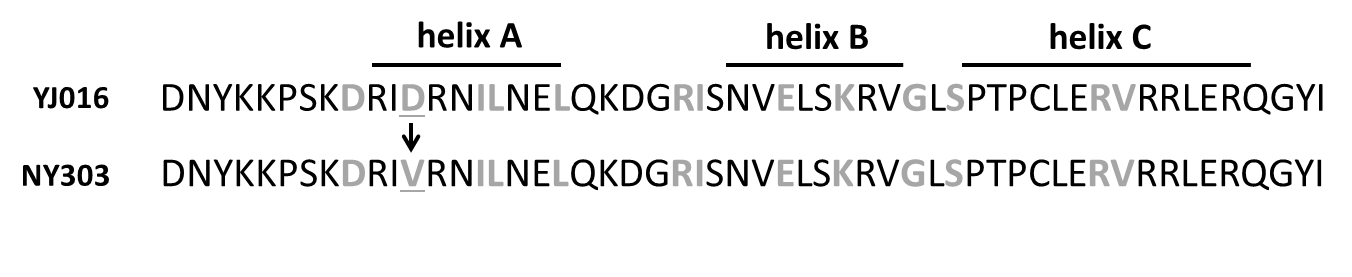


**Fig. S1** Location of point mutation in *lrp* of *V. vulnificus* mutant NY303. **a** The coding sequence of *lrp* contains the HTH ArsR-type DNA-binding domain at N-terminus and AsnC transcriptional regulatory domain at C-terminus for ligand binding (The graph was downloaded from http://www.ncbi.nih.gov). The mutation in *lrp* of mutant NY303 is substitution of A by T at nt 347454 of the genome sequence of strain YJ016 (indicated in gray), which resulted in disruption of the *Cla*I site. **b** Part of the amino acid sequences of strain YJ016 and mutant NY303 are aligned to reveal the point mutation in mutant NY303. This mutation caused change of the 16^th^ residue, which is located within the helix-turn-helix (HTH) domain of Lrp, from aspartate (D) to valine (V) (indicated by an arrow). The highly conserved residues in this region are indicated in gray.
