# Supplementary material for: Lrp, a global regulator, regulates the virulence of Vibrio vulnificus
Source: J Biomed Sci. 2017 Aug 11;24:54. doi: 10.1186/s12929-017-0361-9 (PMC5554404; doi:10.1186/s12929-017-0361-9)
Supplement: Supplementary file 4 — Phagocytosis of various V. vulnificus strains by RAW 264.7 cells after mixed infection. (DOCX 55 kb) [file 12929_2017_361_MOESM4_ESM.docx]

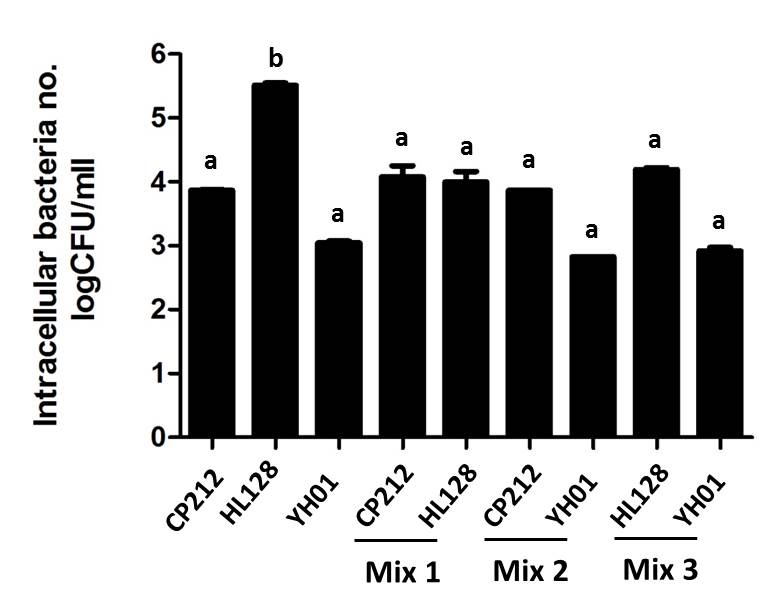


**Fig. S2** Phagocytosis of various *V. vulnificus* strains by RAW264.7 cells after mixed infection. A mixture of equal numbers of two strains was cocultured with RAW264.7 cells in a 96-well microplate at a moi of 10 for 90 min. The intracellular bacterial numbers of each strain were then determined by phagocytosis assay described in ‘Methods’. CP212: YJ016Δ*lacZ* (wild-type strain); HL128: Δ*rtxA1* mutant; YH01: Δ*lrp* mutant. Mix 1: CP212 + HL128; Mix 2: CP212 + YH01; Mix 3: HL128 + YH01. CP212 was distinguished from mutant YH01 or HL128 (white vs. blue colonies) on an X-gal-containing LB plate. Mutant YH01 was distinguished from mutant HL128 (resistant vs. sensitive to chloramphenicol) by growth on chloramphenicol-containing LB plate. Results showing no significant difference are labeled with the same letters, and those showing significant difference (*P* < 0.05) are labeled with different letters based on data analysis by one-way ANOVA followed by Tukey’s test.
