# Supplementary material for: Lrp, a global regulator, regulates the virulence of Vibrio vulnificus
Source: J Biomed Sci. 2017 Aug 11;24:54. doi: 10.1186/s12929-017-0361-9 (PMC5554404; doi:10.1186/s12929-017-0361-9)
Supplement: Supplementary file 5 — The mRNA levels of lrp in the wild-type strain incubated in various media. (DOCX 158 kb) [file 12929_2017_361_MOESM5_ESM.docx]

**a** **b**


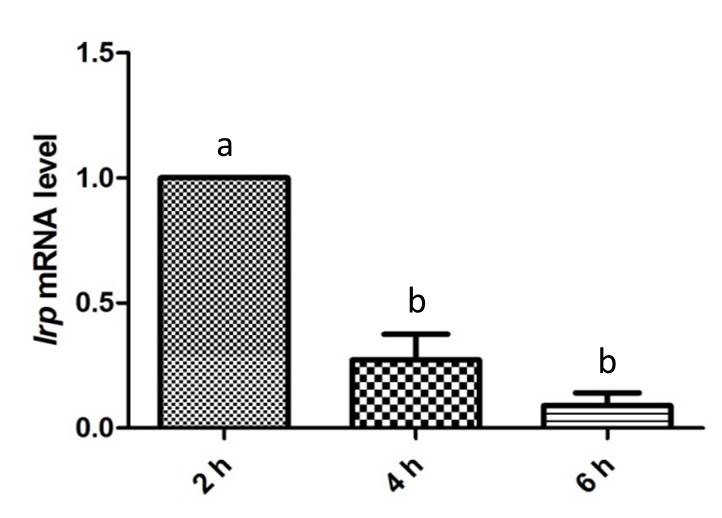

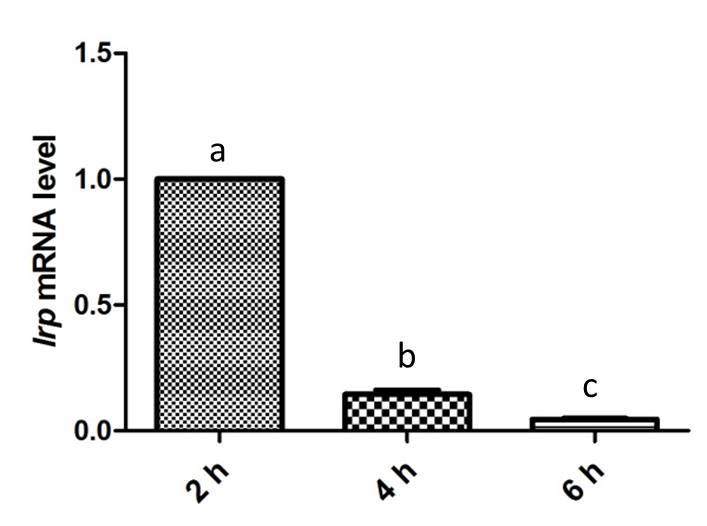


**c** **d**

**
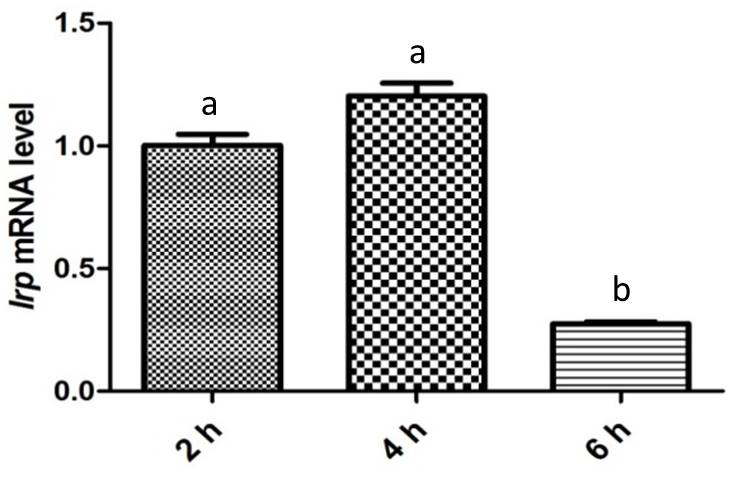
**
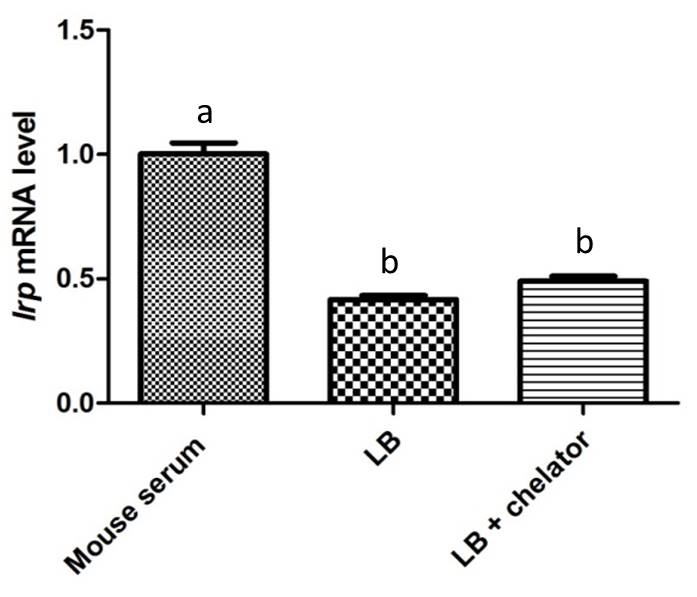


**Fig. S3** The mRNA levels of *lrp* in the wild-type strain incubated in various media. Total RNA collected from a 2 h, 4 h and 6 h cultures of strain YJ016 in LB (**a**), LB containing 100 μM 2,2’-dipyridyl as the chelator (**b**) or 80% mouse serum (**c**) were subjected to qRT-PCR to determine the mRNA levels of *lrp*. **d** A comparison of the *lrp* mRNA levels in stain YJ016 incubated in various media for 2 h. 23S rRNA was used as internal control. n = 3. Results showing no significant difference are labeled with the same letters, and those showing significant difference (*P* < 0.05) are labeled with different letters based on data analysis by one-way ANOVA followed by Tukey’s test.
