# Supplementary material for: Lrp, a global regulator, regulates the virulence of Vibrio vulnificus
Source: J Biomed Sci. 2017 Aug 11;24:54. doi: 10.1186/s12929-017-0361-9 (PMC5554404; doi:10.1186/s12929-017-0361-9)
Supplement: Supplementary file 6 — The distribution of Lrp target genes in different functional categories. (DOCX 334 kb) [file 12929_2017_361_MOESM6_ESM.docx]

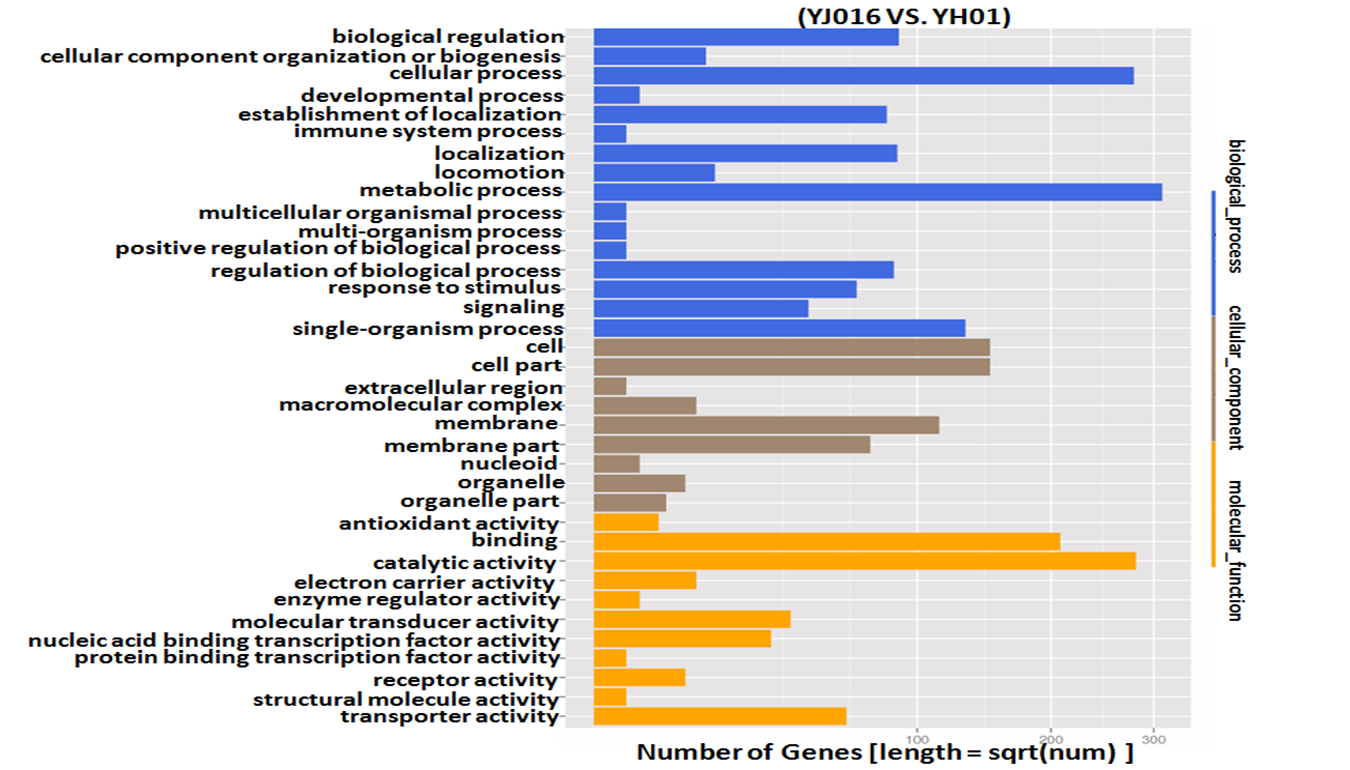


**Fig. S4** The distribution of Lrp target genes in different functional categories. The differentially expressed genes in the Δ*lrp* mutant (YH01) were assigned into different categories of Gene Ontology (GO) as indicated.
