# Supplementary material for: Lrp, a global regulator, regulates the virulence of Vibrio vulnificus
Source: J Biomed Sci. 2017 Aug 11;24:54. doi: 10.1186/s12929-017-0361-9 (PMC5554404; doi:10.1186/s12929-017-0361-9)
Supplement: Supplementary file 7 — The mRNA levels of genes predicted to be involved in iron-acquisition and chemotaxis in the wild-type strain and Δlrp mutant. (DOCX 219 kb) [file 12929_2017_361_MOESM7_ESM.docx]

**a**

**
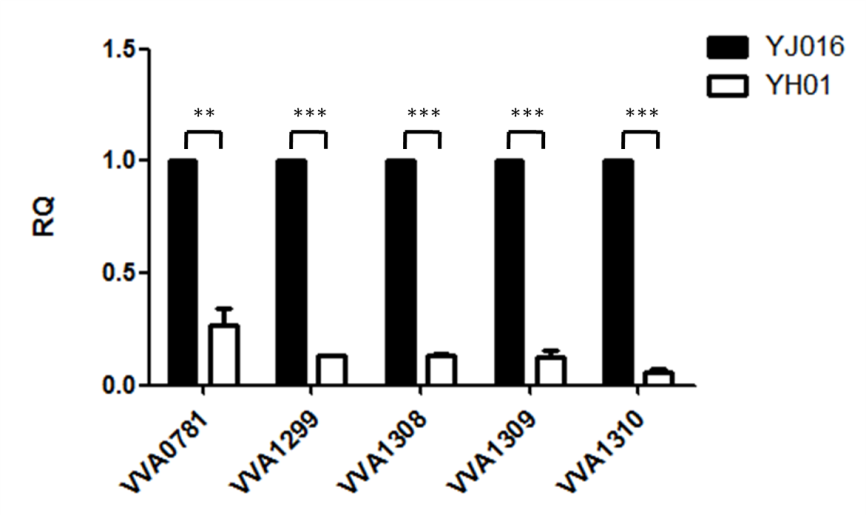
**

**b**


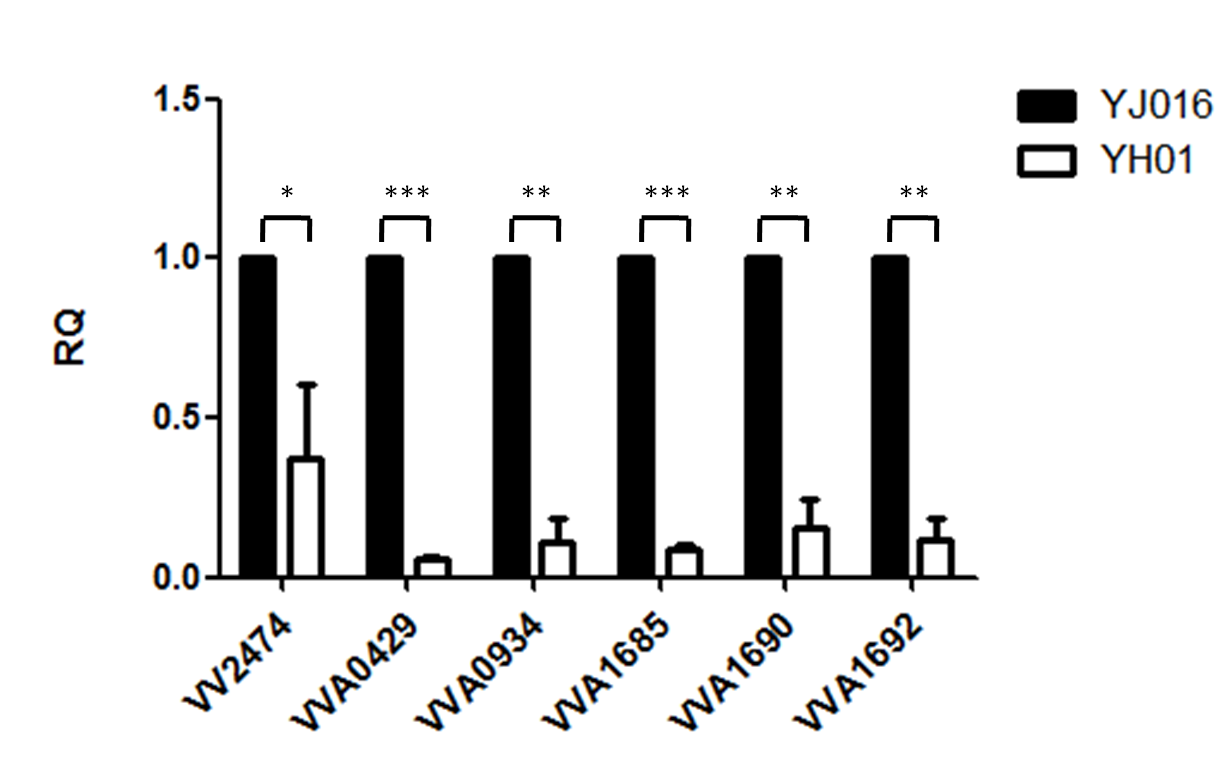


**Fig. S5** The mRNA levels of genes predicted to be involved in iron-acquisition (**a**) and chemotaxis (**b**) in the wild-type strain and Δ*lrp* mutant determined by qRT-PCR. The total RNA samples were extracted from the bacteria incubated in 80% mouse serum for 2 h. RQ: relative mRNA expression. YJ016: wild-type strain, YH01: Δ*lrp* mutant. 23S rRNA was used as an internal control. n=3. The significance of difference was analyzed by t-test. *: *P* < 0.05; **: *P* < 0.01. ***: *P* < 0.001. VVA0781, VVA1299, VVA1308, VVA1309 and VVA1310 in **a** are annotated as heme receptor (HupA), 2,3-dihydroxybenzoate-2,3-dehydrogenase, catechol siderophore ABC transporter; ferric vulnibactin outer membrane receptor, amide synthase subunit of vulnibactin synthetase, respectively. VV2474, VVA0429, VVA0934, VVA1685, VVA1690 and VVA1692 in **b** are annotated as FliM, CheV, CheR, CheB, CheW and CheY, respectively.
